# The DNA Replication Factor *RFC1* Is Required for Interference-Sensitive Meiotic Crossovers in *Arabidopsis thaliana*

**DOI:** 10.1371/journal.pgen.1003039

**Published:** 2012-11-08

**Authors:** Yingxiang Wang, Zhihao Cheng, Jiyue Huang, Qian Shi, Yue Hong, Gregory P. Copenhaver, Zhizhong Gong, Hong Ma

**Affiliations:** 1 State Key Laboratory of Genetic Engineering, Institute of Plant Biology, Center for Evolutionary Biology, School of Life Sciences, Fudan University, Shanghai, China; 2 Department of Biology and the Carolina Center for Genome Sciences, University of North Carolina at Chapel Hill, Chapel Hill, North Carolina, United States of America; 3 Lineberger Comprehensive Cancer Center, University of North Carolina School of Medicine, Chapel Hill, North Carolina, United States of America; 4 State Key Laboratory of Plant Physiology and Biochemistry, College of Biological Sciences, China Agricultural University, Beijing, China; 5 Institutes of Biomedical Sciences, Fudan University, Shanghai, China; CNRS, France

## Abstract

During meiotic recombination, induced double-strand breaks (DSBs) are processed into crossovers (COs) and non-COs (NCO); the former are required for proper chromosome segregation and fertility. DNA synthesis is essential in current models of meiotic recombination pathways and includes only leading strand DNA synthesis, but few genes crucial for DNA synthesis have been tested genetically for their functions in meiosis. Furthermore, lagging strand synthesis has been assumed to be unnecessary. Here we show that the *Arabidopsis thaliana* DNA *REPLICATION FACTOR C1* (*RFC1*) important for lagging strand synthesis is necessary for fertility, meiotic bivalent formation, and homolog segregation. Loss of meiotic *RFC1* function caused abnormal meiotic chromosome association and other cytological defects; genetic analyses with other meiotic mutations indicate that *RFC1* acts in the *MSH4*-dependent interference-sensitive pathway for CO formation. In a *rfc1* mutant, residual pollen viability is MUS81-dependent and COs exhibit essentially no interference, indicating that these COs form via the MUS81-dependent interference-insensitive pathway. We hypothesize that lagging strand DNA synthesis is important for the formation of double Holliday junctions, but not alternative recombination intermediates. That *RFC1* is found in divergent eukaryotes suggests a previously unrecognized and highly conserved role for DNA synthesis in discriminating between recombination pathways.

## Author Summary

Meiotic recombination is important for pairing and sustained association of homologous chromosomes (homologs), thereby ensuring proper homolog segregation and normal fertility. DNA synthesis is thought to be required for meiotic recombination, but few genes coding for DNA synthesis factors have been studied for possible meiotic functions because their essential roles in the mitotic cell cycle make it difficult to study their meiotic functions due to the lethality of corresponding null mutations. Current models for meiotic recombination only include leading strand DNA synthesis. We found that the *Arabidopsis* gene encoding the DNA REPLICATION FACTOR C1 (RFC1) important for lagging strand synthesis promotes meiotic recombination via a specific pathway for crossovers (COs) that involves the formation of double Holliday Junction (dHJ) intermediates. Therefore, lagging strand DNA synthesis is likely important for meiotic recombination. Because DNA synthesis is a highly conserved process and meiotic recombination is highly similar among budding yeast, mammals, and flowering plants, the proposed function of lagging strand synthesis for meiotic recombination might be a general feature of meiosis.

## Introduction

**Figure 1:**
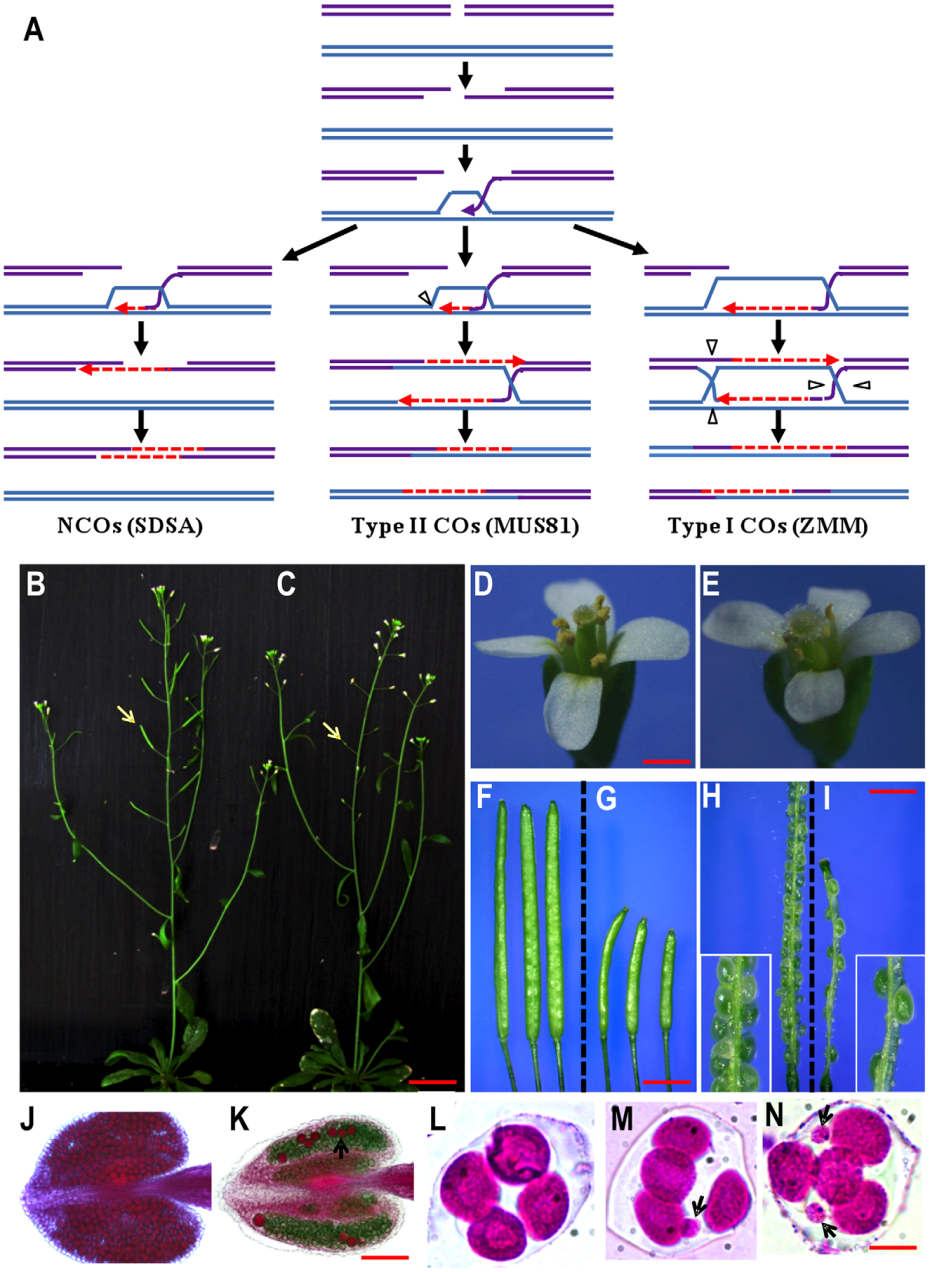
A model for meiotic recombination and phenotypes of wild type and the *rfc1-2* mutant. (A) DNA double-strand breaks are resected to yield 3′ ssDNA overhangs. One of the single strands invades a homologous duplex to form a SEI intermediate. NCOs by the SDSA pathway accounts the majority of DSBs. The ZMM-dependent pathway requires the formation of dHJ and results in ∼80% of COs (Type I), whereas the MUS81-Mms4 dependent pathway produces ∼20% COs (Type II). The formation of both COs and NCOs requires DNA synthesis, but with distinct amounts. (B–N) Phenotypes of wild type and *rfc1-2.* Wild type (B) and *rfc1-2* (C) plants showed similar vegetative growth, but the mutant had shorter seedpods (arrow). A mutant flower (E) had normal organs similar to those in wild type (D). Wild type seedpods (F) and shorter *rfc1-2* mutant seedpods (G). Dissected young wild type (H) and mutant seedpods (I), with very few seeds. The boxed regions were enlarged from H and I. A wild type anther with viable pollen grains stained in red (J); a mutant anther (K) with few viable pollen grains (arrow) and a larger number of nonviable pollen grains stained in dark green. A wild type tetrad with four microspores (L). *rfc1-2* mutant polyads with additional small microspores (arrow) (M and N). Bar = 20 mm (B and C), 500 µm (D and E), 1 mm (F–I), 50 µm (J and K), and 10 µm (L–N).

Meiosis reduces the genomic complement of the cell by half in preparation for fertilization and is essential for sexual reproduction. Recombination is a key event in meiotic prophase I and is important for homolog pairing, bivalent formation and proper homolog segregation [1], [2]. According to the double-strand break repair (DSBR) model [3] (Figure 1A), largely based on molecular studies in yeast and supported by genetic analyses in other organisms [1], [4], meiotic recombination is initiated by SPO11-catalyzed DSBs [5], which are processed to yield 3′ single-strand DNA (ssDNA) overhangs and stabilized by replication protein A (RPA) [6]. RPA is displaced by RecA-like proteins RAD51 and DMC1 to form a nucleoprotein filament, which searches for a homologous template and promotes strand invasion to form a joint molecule in a process called single end invasion (SEI), thereby providing a 3′ end as a primer for DNA synthesis in the nascent D loop [7]. *Arabidopsis thaliana* has five *RPA* homologs, one of them is required for meiotic recombination; unlike the yeast RPA, it likely functions downstream of RAD51 [8]. Subsequently, second DSB end capture results in a double Holliday Junction (dHJ) that is resolved to yield crossovers (COs) and non-crossovers (NCOs) [9], [10]. Alternatively, the invading strand dissociates from the D-loop and re-anneal to the other DSB end to form a NCO via synthesis dependent strand annealing (SDSA) [11].

The formation of both COs and NCOs requires DNA synthesis, but few factors for DNA synthesis have been functionally analyzed in meiotic recombination. In DNA replication, continuous 5′ to 3′ leading strand synthesis requires DNA polymerase (Pol) α-primase to synthesize a short primer and Pol ε, which can sometimes be replaced by Pol δ [12], [13]. Lagging strand synthesis is more complex and requires synthesis and ligation of a series of “Okazaki fragments”, which are initiated from short RNA-DNA primers produced by Pol α-primase [14], [15]. The primer is recognized by the RFC complex, which facilitates the dissociation of Pol α-primase and loading of Proliferating Cell Nuclear Antigen (PCNA). PCNA then recruits Pol δ to the primer-template duplex in a process called ‘polymerase switching’. The discontinuous Okazaki fragments are then processed by DNA endonucleases (such as DNA2 and Fen1) and ligated to complete lagging strand synthesis [14]. In addition to the ATP dependent PCNA loading, RFCs was shown to greatly stimulate Fen1 activity [16], and to participate in the NER (nucleotide excision repair) process [17].

Because many DNA synthesis genes are essential for mitotic growth, null mutants are lethal, precluding the analysis of their meiotic defects. Consequently, the role of DNA synthesis in meiotic recombination has been tested genetically only in a few instances. A four amino acid deletion near the C-terminus of yeast *Pol δ* and *rpa1* mutations in *Arabidopsis* and rice result in meiotic recombination defects [8], [18], [19]. Because both Pol δ and RPA1 are required for leading and lagging strand DNA synthesis, it is not known whether meiotic recombination requires the lagging strand synthesis factors. Indeed, meiotic recombination models include only leading strand synthesis, probably because the amount of leading strand synthesis using the 3′ single-strand invasion end as a primer seems sufficient.

A recent analysis of the *Arabidopsis* male meiocyte transcriptome revealed the expression of several DNA synthesis genes [20]. Among these, the DNA replication factor *RFC1* (the ortholog of the yeast and animal *RFC1* genes) was verified by real-time PCR and *in situ* mRNA hybridization (data not shown), suggesting a function in male meiosis. Here we report a partial loss-of-function *rfc1* mutant that has normal vegetative and floral organ development, but displays reduced fertility and meiotic defects. This *rfc1* mutant provides an opportunity to test the role of lagging strand synthesis in meiotic recombination. Our analyses demonstrate that the *rfc1^−/−^* plants form multivalents and are defective in CO formation via the interference-sensitive pathway (Type I), supporting the idea that lagging strand synthesis is probably important for dHJ formation. Because DNA synthesis is a highly conserved process and the Type I pathway produces the majority of COs in budding yeast, mammals, and flowering plants, a proposed role for lagging strand synthesis in CO formation has important implications for human reproductive health and crop production.

## Results

### *rfc1* mutant alleles

To test *RFC1* function, we obtained three alleles (Figure 1 and Figure S1). *rfc1-1* is a point mutant with defects in vegetative development and somatic DNA repair [21]; it has reduced seed production, sheds both viable and nonviable pollen grains, and suffers from mild meiotic defects (Figure S1E–S1H). *rfc1-2* (SALK_140231) is a T-DNA insertional allele with reduced fertility; *rfc1-3* is also an T-DNA insertional allele but causes seed lethality and can only be maintained as a heterozygote [22]. *rfc1-2^−/−^* has normal leaf number, biomass, floral organ identity and number (Figure 1B–E), indicating that it does not have impaired mitotic DNA replication. However, *rfc1-2^−/−^* plants have greatly reduced fertility (Figure 1C) and produce short seedpods with few seeds (Figure 1G and 1I).

F1 progeny derived from pollinating *rfc1-2^−/−^* pistils with wild type pollen were fully fertile, but mutant and normal plants segregated in the F2 progeny with a ratio of 2.53∶1 instead of the expected ratio of 3∶1 (p<0.05) (Table S1). To verify the decreased transmission of the *rfc1-2* allele, we pollinated *rfc1-2^−/+^* plants with wild type pollen and found ∼50% (293/577) of progeny with the mutant allele (Table S1). When wild type pistils were pollinated with pollen from an *rfc1-2^−^* ^/+^ plant, only 32% (105/329) of the progeny inherited the mutant allele (Table S1), indicating reduced transmission of *rfc1-2* through the male but not the female gametophytes. Additionally, *rfc1-2^−/−^* anthers had few viable pollen grains (∼20/anther; n = 50), in contrast to ∼500/anther in wild type (Figure 1J and 1K). In addition, unlike the wild type tetrads with four microspores (Figure 1L), *rfc1-2^−/−^* anthers contained polyads with five to eight microspores (Figure 1M and 1N), indicating a meiotic defect. Because *RFC1* is essential for the mitotic cell cycle in yeast and is highly conserved in eukaryotes [23], we expected it to be essential in *Arabidopsis* as well. Indeed, *rfc1-3^−/+^* plants produce about 51% (178/349) defective seeds and no homozygous progeny (n = 385), suggesting that seeds homozygous for *rfc1-3* were lethal.

We estimated *RFC1* expression in *rfc1-2^−/−^* plants and found that it was transcribed upstream of, but not spanning the T-DNA insertion (Figure S1C), indicating that a truncated *RFC1* transcript is produced. Probing western blots with a polyclonal antibody against RFC1 revealed a ∼110 kDa band corresponding to the predicted molecular weight of RFC1 in the wild type, but not in *rfc1-2^−/−^* plants (Figure S1D), indicating that *rfc1-2^−/−^* lacked the intact protein.

### RFC1 is required for bivalent formation

**Figure 2:**
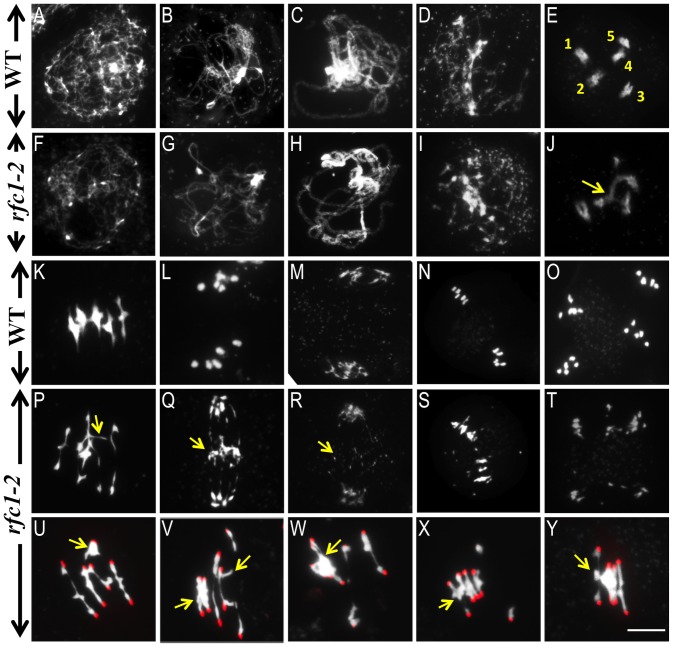
Male meiosis in wild type and *rfc1-2.* (A–D) Wild type chromosome behavior at leptotene, zygotene, pachytene and diplotene, respectively. (F–I) *rfc1-2* chromosome phenotypes were similar to the wild type up to diplotene. (E and J) Wild type diakinesis meiocytes showed five pairs of attached condensed homologs (bivalent, marked with number), but the mutant formed multivalents frequently instead of normal bivalents (arrow points to the association of more than two chromosomes). (K) Five bivalents aligned at the equator at metaphase I in the wild type, but the mutant showed non-homologous interaction (arrow) at a similar stage (P). (L) The homologs were separated by the spindle in wild type after metaphase I. Anaphase I (L and Q), Telophase I (M and R), Metaphase II (N and S) and Telophase II (O and T). The mutant showed chromosome fragmentation from anaphase I to telophase II (arrow). (U–Y) The *rfc1-2* meiocytes formed abnormally long bivalents and multivalents that involved two (U and V), three (W), four (4) and five pairs of chromosomes (Y) (arrow). Red dots represent the centromere signals by FISH. Bar, 10 µm.

The normal mitotic phenotype of *rfc1-2* ^−/−^ suggests replication is not affected; in addition, analysis of meiotic chromosomes in *rfc1-2* ^−/−^ *spo11-1* ^−/−^ (below) indicated that premeiotic replication produced all 20 chromatids. We analyzed male meiosis using chromosome spreads to compare wild type and *rfc1-2* ^−/−^. From leptotene to pachytene, wild type and *rfc1-2* ^−/−^ meiotic chromosome morphologies were generally similar (Figure 2A–2C and 2F–H), but *rfc1-2* ^−/−^ pachytene chromosomes sometimes had small “bubbles” (31%, 26/85), with incomplete synapsis. At diplotene, wild type meiocytes had associated condensed homologs (Figure 2D), but the *rfc1-2* ^−/−^ chromosomes were less compact with regions that were thinner than normal (Figure 2I). At diakinesis, unlike the five intact bivalents in wild type (Figure 2E), *rfc1-2* ^−/−^ meiocytes (n = 76) had 0 (18), 1 (24), 2 (25), 3 (7), or 4 (2) bivalents, and association between more than two chromosomes (Figure 2J). The mean number of bivalents in *rfc1-2* ^−/−^ was 1.36. At metaphase I, the mutant bivalents were often slender with only one crossover (Figure 2P). In addition, four or more *rfc1-2* ^−/−^ chromosomes often formed multivalents in which one chromosome was associated with two other chromosomes (Figure 2U–2V). At anaphase I and later stages, chromosome fragmentation was observed (Figure 2Q–2T). The defects in bivalent formation and chromosome segregation are consistent with the formation of polyad microspores and reduced pollen viability.

To verify that the *rfc1-2* ^−/−^ phenotype is caused by the T-DNA insertion in the *RFC1* gene, we introduced a wild type *RFC1* gene into the *rfc1-2* mutant and rescued the mutant fertility and meiosis defects (Figure S2A–S2D). To test *RFC1* function in meiosis using plants with even less RFC1 activity than the *rfc1-2* allele, we performed reciprocal crosses between *rfc1-2* ^−/+^ and *rfc1-3* ^−/+^ and identified *rfc1-2/rfc1-3* trans-heterozygous progeny using PCR. The trans-heterozygote was very small, suggesting a defect in mitotic growth, unlike *rfc1-2* ^−/−^ plants; nevertheless, the *rfc1-2/rfc1-3* meiocytes showed meiotic phenotypes similar to *rfc1-2* ^−/−^ (Figure S2E–S2H). We also generated transgenic plants with an RNAi construct of *RFC1* driven by the meiosis-specific *DMC1* promoter and obtained similar meiotic phenotypes to *rfc1-2* ^−/−^ mutants (Figure S2I–S2L), even when pollen viability and fertility was more severely affected than in *rfc1-2* ^−/−^. These results demonstrate that lesions in *RFC1* cause the meiotic and fertility defects, and also indicate that the meiotic phenotypes in *rfc1-2* ^−/−^ plants are due to a meiosis-specific loss-of-function, rather than the effect of a toxic protein.

### RFC1 is required for homolog pairing and synapsis

**Figure 3:**
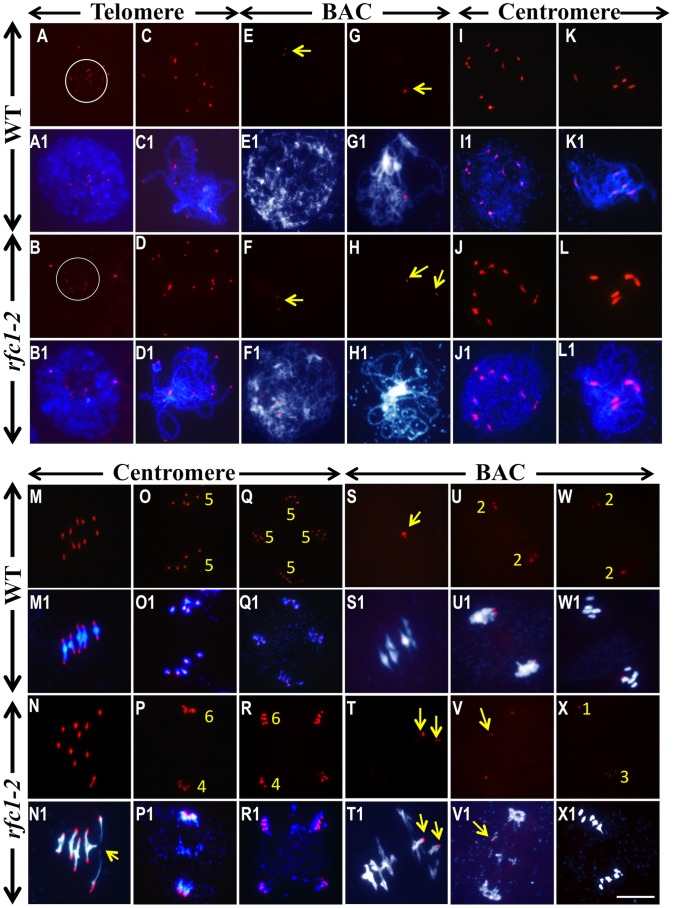
FISH analysis of the chromosome interaction in wild type and *rfc1-2.* (A) Wild type and (B) *rfc1-2* interphase nuclei with telomere clusters (circled). (C) Wild type and (D) *rfc1-2* pachytene cells with telomere signals. (E) Wild type and (F) *rfc1-2* leptotene cells with two chromosome 1 BAC F19K16 signals (arrow). (G) A wild type pachytene cell with one signal, and a mutant pachytene cell (H) with two separated signals (arrow). (I, K, M, O, Q) Wild type and (J, L, N, P, R) *rfc1-2* meiocytes with a centromere probe. Leptotene with 10 signals (I1 and J1) and pachytene with five signals (K1 and L1). At Metaphase I, wild type had 10 signals on five bivalents (M1), but *rfc1-2* showed non-homolog association (N1, arrow), At anaphase I and telophase II, wild type had 5 signals in each group (O1 and Q1), but *rfc1-2* showed chromosome missegregation with 4 or 6 signals in each group (P1 and R1). Wild type (S) had one BAC F19K16 signal at metaphase I, but *rfc1-2* (T) had two signals (arrow). At anaphase I and II, wild type (U and W) had two signals on each side, but *rfc1-2* (V and X) showed chromosomes missegregation with unequal signals (arrow). Red dots represent the signals of different probes. The superimposed images were produced by merging FISH signals with DAPI-stained chromosomes. Bar, 10 µm.

To test whether the *rfc1-2* ^−/−^ bivalents occured between homologs or non-homologs, we used FISH to analyze chromosome pairing and synapsis. In several species, telomeres cluster on the nuclear envelope during leptotene/zygotene in a configuration called a “bouquet”, which facilitates pairing and synapsis [24]. *Arabidopsis* appears to lack a classic bouquet but is thought to achieve the same function by clustering telomeres around the nucleolus before leptotene [25]. We observed a similar pattern in both wild type and *rfc1-2* ^−/−^ mutant spreads (Figure 3A–3B) and *rfc1-2* ^−/−^ samples were also normal at zygotene (data not shown). At pachytene, wild type cells had 8–10 telomere foci (n>20), consistent with five pair of closely associated homologs (Figure 3C); however, ∼45% *rfc1-2* ^−/−^ mutant cells (n = 30) had more than 10 (but fewer than 20) foci (Figure 3D), indicating partial separation of telomeres.

To test pairing defects in *rfc1-2* ^−/−^ plants, we performed chromosome-specific FISH using a BAC clone (F19K16) located in a telomere-proximal region of chromosome 1. Similar to the telomere result, the F19K16 signals in wild type and mutant were similar at leptotene and zygotene (Figure 3E and 3F; Figure S3). At pachytene, wild type had only one signal representing the closely synapsed chromosome 1 arm (Figure 3G), but ∼40% of the *rfc1-2* ^−/−^ cells (n = 43) had two signals (Figure 3H), indicating that the two chromosome 1 arms were at least partially separated. The observation of two separate F19K16 signals in the thin chromosome regions further indicated that the homologs were not properly paired or synapsed at this region in the mutant (Figure S3E and S3G). FISH with a centromere probe revealed the same number of signals between wild type and mutant at leptotene and pachytene (n>60) (Figure 3I–3L), indicating that the mutant had no obvious pairing and synapsis defects near the centromeres, similar to the results with a 45S rDNA probe (Figure S3I–S3T).

At diplotene, wild type cells had pairs of closely spaced F19K16 signals corresponding to partially separated homologs (Figure S3B), whereas more widely spaced F19K16 signals were observed in mutant cells (∼50%, n = 40) (Figure S3D). Wild type and mutant metaphase I cells both had 10 centromere signals (Figure 3M and 3N); however, mutant cells often had multivalents with four or more chromosomes (Figure 3N1), indicating non-homologous association. In addition, wild type had only one F19K16 signal (Figure 3S), indicating close association of the two chromosome 1's, but the mutant often had two foci (Figure 3T), suggesting that two chromosome 1's had associated with non-homologs. At anaphase I, wild type chromosomes segregated equally with 5 centromeric foci in each group (Figure 3O and Q), but mutant chromosomes often missegregated (Figure 3P and 3R). Acentric chromosome fragments, including some with F19K16 foci, were seen near the equator (Figure 3P and 3R–3V), possibly resulting from illegitimate non-homologous recombination. Similar results were also obtained using another BAC probe F1N21 (not shown).

**Figure 4:**
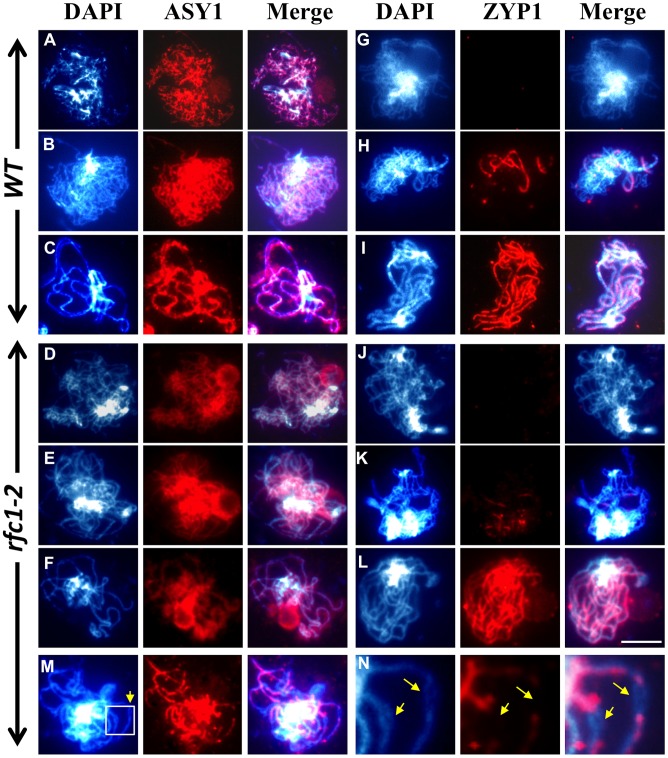
Immunolocalization of the ASY1 and ZYP1 proteins in wild type and *rfc1-2.* (A–F) Localization of ASY1 was similar between wild type and mutant, respectively, at leptotene (A and D), zygotene (B and E) and pachytene (C and F). Localization of ZYP1in wild type (G–I) and *rfc1-2* (J–N) at leptotene (G and J), zygotene (H and K) and pachytene (I and L–N). (M and N) *rfc1-2* pachytene chromosomes had a “bubble” region lacking the ZYP1 signal (arrow). N is enlarged region from M. In each row of three panels, the left panel shows blue colored chromosomes strained with DAPI; the middle panel shows red colored signals for proteins as indicated above the panel; and the right panel shows the merged image of DAPI and protein signals. Bar, 10 µm (A–M).

To further analyze synapsis in *rfc1-2* ^−/−^, we examined the distribution of ASY1 and ZYP1, which mediate formation of the synaptonemal complex (SC) [26], [27]. Immunolocalization of both proteins is similar in wild type and *rfc1-2* ^−/−^ from leptotene to pachytene (Figure 4A–4L), except that *rfc1-2* ^−/−^ pachytene chromosomes sometimes had a “bubble” lacking the ZYP1 signal (Figure 4M and 4N). Therefore, axis formation as indicated by the signal of the lateral element protein ASY1 was normal in *rfc1-2* ^−/−^, but the lack of signal for the central element protein ZYP1 in the “bubble” regions suggested a partial defect in synapsis.

### RFC1 functions in the SPO11-1-dependent pathway and acts downstream of RAD51

**Figure 5:**
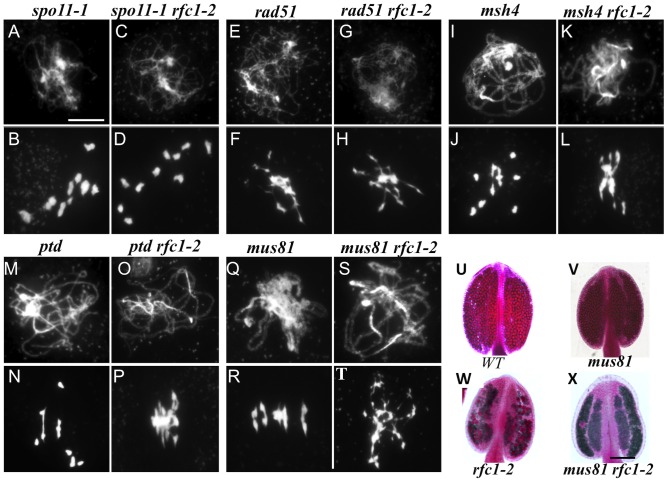
Genetic analysis of *RFC1* with other meiotic recombination genes. Chromosome behavior of male meiocytes at pachytene and metaphase I. (A–B) *spo11-1-1^−/−^*. (C–D) The *spo11-1-1^−/−^ rfc1-2^−/−^* double mutant showing similar chromosome behaviors to those of the *spo11-1-1^−/−^* single mutant. (E–F) *rad51-3^−/−^*. (G–H) The *rad51-3^−/−^ rfc1-2^−/−^* double mutant showing similar phenotypes to those of the *rad51-3^−/−^* single mutant. (I–J) *msh4-1^−/−^*. (K–L) The *msh4-1^−/−^ rfc1-2^−/−^* double mutant showing similar phenotypes to those of the *rfc1-2^−/−^* single mutant. (M–N) *ptd.* (O–P) The *ptd-1^−/−^ rfc1-2^−/−^* double mutant showing similar to the *rfc1-2^−/−^* single mutant. (Q and R) *mus81.* (S–T) The *mus81-1^−/−^ rfc1-2^−/−^* double mutant lacking multivalent, unlike the *rfc1-2^−/−^* single mutant. (U–X) Viability of pollen grains in wild type (U), *mus81-1^−/−^* (V), *rfc1-2^−/−^* (W) and *mus81-1^−/−^ rfc1-2^−/−^* (X), the *mus81-1^−/−^ rfc1-2^−/−^* double mutants showing almost no viable pollen grains. Bar, (A–T) 10 µm, (U–X) 500 µm.

The *rfc1-2* ^−/−^ abnormalities in pairing and bivalent formation suggest possible defects in meiotic recombination. In yeast, meiotic recombination is initiated by SPO11-generated DSBs [5] (Figure 1A) and one of the *Arabidopsis* homologs, *SPO11-1*, is also important for DSB formation, chromosome pairing and bivalent formation [28]. RAD51 in yeast is crucial in homolog dependent single strand invasion [5] (Figure 1A) and its *Arabidopsis* homolog (*RAD51*) is required to process SPO11-1 induced DSBs [29]–[30]. Failure to repair SPO11-1-induced DSBs in *rad51* and other mutants results in chromosome fragmentation, which is absent in the *spo11-1 rad51* double mutant [30]. Because RFC1 is a DNA synthesis factor and DNA synthesis is proposed to occur after RAD51-dependent single strand invasion and the formation of SEI, we hypothesized that chromosome fragmentation in *rfc1-2* ^−/−^ depends on SPO11-1 and that *rfc1-2* ^−/−^ could not suppress the defects of *rad51.* To test our hypotheses, we found that the meiotic prophase I of *spo11-1-1* ^−/−^ *rfc1-2* ^−/−^ double mutant (Figure 5C–5D) was similar to that of the *spo11-1-1* ^−/−^ with many univalents (Figure 5A–5B), but different from that of *rfc1-2* ^−/−^ (Figure 2J and 2P). To test for epistasis of *rad51-3* ^−/−^ and *rfc1-2* ^−/−^, chromosome spreads from *rad51-3* ^−/−^ were prepared and lacked bivalents or multivalents (Figure 5E–5F), but *rfc1-2* ^−/−^ had both (Figure 2J and 2P). The *rad51-3* ^−/−^ *rfc1-2* ^−/−^ double mutant resembled *rad51-3* ^−/−^, without any multivalents (Figure 5G–5H), indicating that *rad51* is epistatic to *rfc1-2* ^−/−^. Therefore, these results support the hypothesis that RFC1 acts after RAD51 to promote recombination.

**Figure 6:**
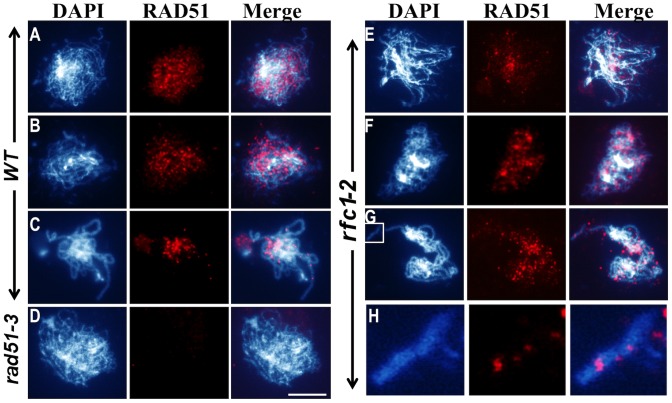
RAD51 localization in wild type and *rfc1-2.* (A–H) Localization of RAD51. (A and E) leptotene, (B and F) zygotene, (C, D and G) pachytene, (H) An enlarged image from (G), (D) the *rad51* mutant. The *rfc1-2* mutant showed RAD51 foci highly similar to the wild type ones before pachytene, but with persisted signals at pachytene (G). In each row of three panels, the left panel shows blue colored chromosomes strained with DAPI; the middle panel shows red colored signals for proteins as indicated above the panel; and the right panel shows the merged image of DAPI and protein signals. Bar, 10 µm.

If RFC1 acts downstream of RAD51, it should not be required for RAD51 loading onto meiotic chromosomes. Immunofluorescence showed that RAD51 foci were similar in wild type and *rfc1-2* ^−/−^ at leptotene (Figure 6A and 6E). At zygotene and pachytene wild type had an average of 230±51 (n = 30) and 50±12 (n = 35), RAD51 foci respectively (Figure 6B and 6C). *rfc1-2* ^−/−^ meiocytes had similar numbers of RAD51 foci (241±55, n = 30) at zygotene (Figure 6F) (p>0.05), indicating that RAD51 loading is not compromised in *rfc1-2* ^−/−^. However, unlike the reduction of RAD51 foci from late zygotene to late pachytene in the wild type, the RAD51 foci persisted in *rfc1-2* ^−/−^ (220±50 foci, n = 39) at pachytene (p<0.001; Figure 6G). This suggests that in *rfc1-2* ^−/−^ RAD51unloading is delayed or new RAD51 foci were generated during pachytene. In addition, wild type DMC1 signals were punctate at pachytene (Figure S4C), but in *rfc1-2* ^−/−^ there were more signals that were distributed along the chromosomes (Figure S4G–S4H). The persistence of RAD51 and DMC1 foci in *rfc1-2* ^−/−^ suggested that RFC1 is important for the normal processing of RAD51/DMC1-bound single-end invasion (SEI) intermediates or RFC1-dependent intermediates are important for the homeostasis of DSB formation.

### RFC1 is important for CO formation via the interference-sensitive pathway

*Arabidopsis*, like yeast and humans, has at least two distinct classes of COs [31]–[33]. Interference-sensitive COs (Type I) require MSH4/MSH5 and PTD [34], [35], whereas interference-insensitive COs (Type II) depend on MUS81 [36]. To determine whether RFC1 affects one or the other of the two CO pathways, we generated double mutants with *rfc1-2* ^−/−^ and examined chromosome spreads. We found that *msh4-1* ^−/−^ *rfc1-2* ^−/−^ (Figure 5K–5L) and *ptd-1* ^−/−^ *rfc1-2* ^−/−^ (Figure 5O–5P) double mutants had phenotypes of chromosome entanglement and fragmentation similar to those of the *rfc1-2* ^−/−^ single mutants. The mean numbers of bivalents of the double mutant *msh4-1* ^−/−^ *rfc1-2* ^−/−^ (n = 18) and *ptd-1* ^−/−^ *rfc1-2* ^−/−^ (n = 19) were 1.27 and 1.47, respectively. These results suggest that RFC1 acts upstream of MSH4 and PTD in the Type I CO pathway. In contrast, the *mus81-1* ^−/−^ *rfc1-2* ^−/−^ double mutant lacked bivalents or multivalents at metaphase I, but had entangled chromosomes (Figure 5S–5T), suggesting that formation of multivalents or chromosome association in *rfc1-2* ^−/−^ also depends on MUS81. This idea is further supported by the observation that residual viable pollen grains in *rfc1-2* ^−/−^ are virtually eliminated in the *mus81-1* ^−/−^ *rfc1-2* ^−/−^ double mutant. Wild type, *mus81-1* ^−/−^ and *rfc1-2* ^−/−^ anthers (n = 50) had approximately 500, 350 and 20 viable pollen grains, respectively (Figure 5U–5W), while *mus81-1* ^−/−^ *rfc1-2* ^−/−^ anthers contained no viable pollen, with only occasional abnormally large pollen grains (Figure 5X). These results indicate that RFC1 likely acts in the same pathway as MSH4 and PTD.

**Figure 7:**
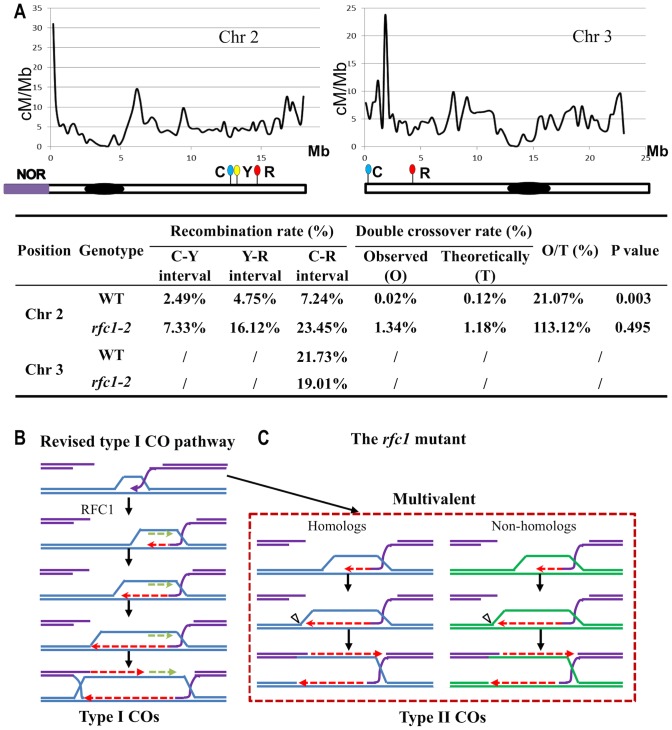
Meiotic recombination rate in *rfc1-2* and a revised model for RFC1 in meiotic recombination. A. Estimation of the recombination rate in wild type and *rfc1-2*. Curves in graphs indicate the distribution of meiotic recombination frequency on *Arabidopsis* chromosome 2 and 3 [38]. Markers with red (R), cyan (C) and yellow (Y) fluorescence are located on the corresponding chromosome positions. The recombination rates in wild type and mutant are shown in the table below. The same trend was obtained from two independent biological replicates. NOR, Nucleolar organizer region; Chr, chromosome. Block box represents the centromere. B. A revised model for RFC1 function in type I CO formation during meiotic recombination. As shown in Figure 1A or original DSBR model [3], NCOs and COs are proposed to require leading strand DNA synthesis. However, formation of dHJ requires the capture of the second ssDNA end and the stabilization and extension, respectively, of the DNA heteroduplex with the help of the MSH4/5 complex and MER3. In the revised model, we hypothesize that the Type I pathway needs the RFC1-dependent lagging strand DNA synthesis (green dotted arrow) during D-loop extension, which occurs simultaneously with leading strand synthesis (red dotted arrows) that uses the 3′ invading end as the primer, consistent with a DNA replication mechanism in eukaryotes and supported by this study. C. A possible mechanism for the formation of multivalents in *rfc1-2*. RFC1 is required for dHJ formation between homologs. In the absence of RFC1, MUS81-dependent Type II COs are formed between homolog and non-homologs, resulting in the formation of multivalents.

We hypothesize that the diminished pollen viability of *rfc1-2* ^−/−^ is caused by a reduction of COs. The residual viable pollen in *rfc1-2* ^−/−^ allowed us to measure CO frequency using a unique pollen-based visual assay system [37]. We crossed *rfc1-2* ^−/+^ with two lines each carrying two or three transgenic markers on chromosomes 2 or 3 encoding different fluorescent proteins (CFP, YFP and RFP) (Figure 7A), which are expressed from the pollen-specific *LAT52* promoter so their expression in the pollen is directly correlated with the segregation of the markers. As a result, it is possible to distinguish using fluorescence microscopy whether linked markers have experienced an intervening CO by analyzing F2 individuals that were heterozygous for the markers and either *RFC1* wild type or *rfc1-2^−/−^*.

Because COs facilitate proper homolog segregation, the recombination frequency from the small number of viable pollen of *rfc1-2* ^−/−^ is expected to be an over-estimate of the overall frequency among all pollen grains, most of which were dead. CO frequency in the *Arabidopsis* genome is uneven with hot and cold regions [38] (Figure 7A). We found that for a pair of markers in a hot region on chromosome 3, the frequency in *rfc1-2* ^−/−^ was lower (19.01%) than wild type (21.73%) (p≪0.001) (Figure 7A, Table S2). In contrast, in a chromosome 2 cold region with three markers, the recombination frequencies in the viable *rfc1-2* ^−/−^ pollen between any two markers were higher than observed in the wild type (Figure 7A and Table S2). The frequency of double COs (one in each adjacent interval) in *rfc1-2* ^−/−^ was 1.34%, and did not differ significantly (p = 0.50) compared to the expected value of 1.18% derived from the product of the individual CO frequencies in the adjacent intervals. However, it was higher than the observed double CO frequency of 0.02% in the wild type (p≪0.001) (Figure 7A and Table S2). These results suggest that wild type COs in this region were largely Type I, but COs in the *rfc1-2* ^−/−^ were Type II.

## Discussion

### RFC1 is important for meiotic homolog association

Mutations in genes encoding the RFC complex cause lethality in mammals and yeast [39], [40], making it difficult to study their functions in meiosis. Similarly, the *Arabidopsis rfc1-3* mutation is also lethal, consistent with a conservation of RFC1 function for mitotic DNA replication in plants. Furthermore, Liu et al. (2010) identified a point mutation in *RFC1* as a suppressor of *ros1* and showed that *rfc1-1* ^−/−^ is small, flowers early and has reduced fertility, indicating that RFC1 is important for *Arabidopsis* vegetative and reproductive development. This approach also identified similar roles for other components of the DNA replication machinery, such as polymerases α, δ, ε and RPA2A [41], suggesting that the DNA replication machinery is essential for vegetative somatic development in plants.

In this study, we have characterized the *rfc1-2* mutant, which has normal vegetative and floral organ development, but has defects in fertility and meiosis. The observations that *rfc1-2* is recessive and can be rescued by wild type trans-complementation, and that both the *rfc1-2/rfc1-3* trans-heterozygote and *RFC1-RNAi* transgenic lines had similar meiotic defects indicate that *rfc1-2* is a meiosis-specific hypomorphic allele. The *rfc1-2* ^−/−^ phenotypes of reduced pollen viability and fertility are the result of reduced bivalents, multivalent formation, and chromosome fragmentation, as supported by FISH using two chromosome-specific BACs. Proper homolog association depends on repair of SPO11-induced DSBs to produce COs, which in turn require DNA synthesis. It is likely that the reduction of RFC1 function during meiosis blocked normal homolog association. The importance of DNA synthesis for meiotic recombination is also supported by the observation in budding yeast that a non-lethal allele of *POL3* has meiotic recombination defects, but normal mitotic growth [19].

Normal meiotic homolog interactions include pairing, synapsis and recombination. Synapsis in *rfc1-2* ^−/−^ occurred, but was sometimes incomplete. Double mutant analysis suggested that RFC1 is in the SPO11-1-dependent pathway, as supported by the observation that the meiotic chromosome fragmentation in *rfc1-2* ^−/−^ depends on SPO11-1-generated DSBs; furthermore, RFC1 probably acts downstream of RAD51, as it is not required for the loading of RAD51 or DMC1 onto chromosomes. The prolonged localization of RAD51 and DMC1 foci at late pachytene in *rfc1-2* ^−/−^ suggest that RFC1 may promote the dissociation of RAD51 and DMC1 [42], [43]. RFC1 likely acts in the same meiotic recombination pathway as several known genes, such as the MRN complex, *COM1*, *RAD51*, *BRCA2* and *MND1/HOP2* [1], [4]. *rfc1-2* ^−/−^ differs from mutants of these genes in having paired homologs with some “bubbles” and some discontinuous ZYP1 at pachytene.

### RFC1 promotes CO formation via the interference-sensitive pathway

Crossover interference influences the distribution of COs in most organisms. Several species, including *Arabidopsis*, budding yeast, mouse and humans have two classes of crossovers: Type I COs are sensitive to interference and Type II COs are not [31]–[33]. The Type I pathway depends on the ZMM proteins (such as MSH4 and MER3), PTD, and MLH1/3 [4], [34], [35], whereas the Type II pathway is typically a minority class in these organisms and depends in part on the Mus81-Mms4 endonuclease [31], [36], . The idea that *rfc1-2* ^−/−^ affected Type I CO formation, but not Type II is supported by several lines of evidence: (1) *rfc1-2* ^−/−^ has reduced bivalent formation, similar to *msh4* ^−/−^ and *ptd* ^−/−^, but more severe than *mus81* ^−/−^; (2) *rfc1-2* ^−/−^ double mutants with *msh4* ^−/−^ or *ptd* ^−/−^ are more similar to *rfc1-2* ^−/−^ single mutants, but *rfc1-2* ^−/−^ *mus81* ^−/−^ is more severe than either *rfc1-2* ^−/−^ or *mus81* ^−/−^; (3) COs in *rfc1-2* ^−/−^ showed no interference.

In yeast meiosis, the Msh4/5 complex is thought to stabilize the crossover specific SEI and subsequently binds to dHJ [45], [46]. The meiosis-specific MER3 helicase promotes RAD51-mediated D-loop extension, and a defect in MER3 causes dramatically delayed homolog alignment accompanied by persistent RAD51 signals [47], [48]. Similarly, *rfc1-2* ^−/−^ also had a large number of persistent RAD51 and DMC1 foci, suggesting that their processing was delayed. It is possible that RFC1 promotes the dissociation of RAD51 and DMC1 and processing of the SEI intermediates to form dHJs, with the help of MSH4 and MER3. The loss of RFC1 function causes a failure to dissociate RAD51 and DMC1, thereby blocking the Type I pathway for CO formation. Instead, the Type II COs are formed between homologs and non-homologs. Alternatively, continued RAD51 and DMC1 foci in late pachytene could be a result of the generation additional DSBs when fewer Type I COs are formed in the *rfc1* mutant, as reported for yeast and mouse meioses [42], [43].

### A revised model for meiotic recombination

As shown in Figure 1A, in classical and revised DSB repair models, DNA synthesis is essential for both CO or NCO pathways and is initiated from single-stranded DNA (ssDNA) generated by 5′-3′ resection of the DNA ends. RAD51 binds to the resulting ssDNA tails to initiate pairing and strand invasion of homologous duplex DNA. Most SEI intermediates form NCOs, and a few form COs. The invading single strand DNA serves as primer for leading strand DNA synthesis before the recombination pathways diverge.

The idea that the amount of DNA synthesis is different between these pathways is strongly supported by a recent analysis using BrdU incorporation [49]. Furthermore, genome-wide analysis in yeast and *Arabidopsis* indicate that the conversion tracts associated with CO are much longer than those associated with NCO [50]–[52], consistent with more extensive synthesis during CO formation. *RFC1* is a single-copy gene in animals, fungi and plants and plays essential roles in loading Pol α-primase for the RNA-DNA primer synthesis, which occurs repeatedly for the production of Okazaki fragments in lagging strand synthesis. Although primer synthesis is also needed to initiate replicative leading strand synthesis, it is not needed for the continuation of leading strand synthesis. Furthermore, as described earlier, leading strand synthesis in meiotic recombination uses the invading strand as the primer, bypassing the need for primer synthesis. Therefore, we propose that *RFC1* is important for lagging strand synthesis during dHJ formation during meiotic recombination (Figure 7B).

The results from *rfc1* ^−/−^ and *RFC1* RNAi plants, as well as double mutants with *msh4* ^−/−^ or *ptd* ^−/−^, indicate that *RFC1* is important for CO formation via the Type I pathway. Furthermore, *rfc1* ^−/−^ mutant phenotypes and genetic analysis with *mus81* ^−/−^ indicate that *RFC1* is not required for the Type II pathway. It is possible that the more extensive DNA synthesis in dHJ formation require synthesis on both strands, as is true for the coordinated synthesis on both strands during replication [13]. If there is only leading strand synthesis, an extended region of single-strand DNA would form on the non-template strand of the duplex DNA, which might be unstable and deleterious. In contrast, lagging strand synthesis results in both sides being double-strand, promoting longer DNA synthesis in a way similar to DNA replication and possibly rendering leading strand synthesis unnecessary after second-end capture.

In yeast, DSBs are directed to either CO or NCO repair early during recombination, before SC formation [53]. Our analysis of *RFC1* function suggests that it plays an early role in promoting dHJ formation by facilitating more extensive DNA synthesis. According to the “ends-apart” model, one DSB end pairs with a homolog chromatid with subsequent DNA synthesis and dHJ formation, whereas the other end remains associated with its sister chromatid [7], [54]. *rfc1-2* ^−/−^ pachytene chromosomes sometimes had “buddles”, suggesting incompleting synapsis, supporting that idea that dHJs facilitate synapsis [53]. It is possible that more DNA synthesis from both leading and lagging strands results in more stable joint molecules that allow the loading of SC proteins. In the absence of RFC1-dependent dHJs, only MUS81-dependent Type II COs are formed, without homolog bias, as non-homologous multivalents were observed (Figure 7C). Alternatively, recombination with a sister chromatid is possible, as seen in yeast [55]. Recently two groups both demonstrated that aberrant joint molecules formed in the yeast *rmi1* or *top3* mutants and were resolved by either SGS1 or Mus81-Mms4 [56]–[58]. Moreover, two studies showed recently that the *Arabidopsis* homolog of the human *Fanconi anemia complementation group M (FANCM)* gene was not only required for the Type I CO formation, but also necessary for the increased number of COs in *msh4* ^−/−^ via the MUS81-dependent pathway [59], [60]. Because the Mus81-Eme1 or Mms4 function is conserved in yeast [44], animal [61], and *Arabidopsis* [62], those SEIs that failed to form dHJs in the *rfc1-2* ^−/−^ or *fancm* ^−/−^ mutants were likely processed by MUS81-dependent activities.

In summary, we have demonstrated that the highly conserved DNA replication factor *RFC1* is required for meiotic recombination and CO formation, and for normal pollen viability and male fertility. Defects in *RFC1* function cause abnormal CO formation, which is dependent on *MUS81* function. Our results provide strong evidence that lagging strand DNA synthesis is critical for the formation of interference-sensitive COs in *Arabidopsis* and we offer a model in which RFC1 mediated lagging strand synthesis promotes CO formation via dHJs.

## Materials and Methods

### Plant material and genotyping

The mutant materials were described as referenced: *rfc1-1* and *rfc1-2* (SALK_140231) [21], *rfc1-3* (SALK_146845), *spo11-1* (SALK_045787), *rad51-3* (SAIL_873_C08) [29], *msh4-1* (SALK_136296) [35], *mus81-1* (SALK_107515) [36] and *ptd-1* (SALK_127447) [34]. Double mutants were identified in the F2 generation by PCR using primers as described in Table S3. Wild type was Columbia (Col-0) except for *rfc1-1*, which was in the C24 background. Plants were grown in a greenhouse under constant 22°C with 16 hr light/8 hr dark.

### Phenotypic analysis

Plants were photographed with a Canon digital camera (Canon, Tokyo, Japan). Pollen grains were stained with Alexander red. Dissected tetrads were stained with 0.01% fuchsin basic. Chromosome spreads of wild type and mutants were prepared as described previously [63] and stained with 1.5 µg/ml 4,6-diamidino-2-phenylindole (DAPI). Images of chromosome spreads were obtained using a Zeiss Axio Imager A2 microscope (Zeiss, Heidelberg, Germany). The images were organized using Photoshop CS (Adobe Systems, Mountain View, CA).

### Western blot

The total proteins from inflorescences were prepared as described previously [64]. The protein amount was estimated with a Bio-Rad Protein Assay Kit as specified by the manufacturer (Bio-Rad, Berkeley, CA, USA). The extraction buffer was supplemented with a protease inhibitor cocktail (catalog No. 1836170; Roche, Mannheim, Germany). Proteins were separated on 10% SDS-PAGE and transferred onto PVDF membranes (catalog No. RPN1416F; Amersham Biosciences, Piscataway, NJ, USA). The membranes were blocked overnight at 4°C in TBST (TBS plus 0.05% Tween20) containing 5% defatted milk, and incubated in polyclonal anti-RFC1 antibody at a final concentration of 0.1 µg/ml. After washed in TBST, the membranes were incubated with 1∶2,000 dilution of peroxidase-conjugated goat anti-rabbit antibody (catalog No. 03116930001; Roche Diagnostics, Indianapolis, Indiana, USA) for 1 hr and then washed thoroughly. Immunoblotting bands were detected using the ECL PlusTM Reagents (catalog No. RPN2132; Amersham Biosciences) according to the manufacturer's protocol.

### Constructs and plant transformation

To rescue the *rfc1-2* mutant, the full-length cDNA of *RFC1* was amplified using primers (oMF-1000 and oMF-1001) with KOD plus DNA polymerase (Toyobo, Tokyo, Japan). The PCR product was purified and ligated into *pEASY*-T1 Simple (Transgen, Beijing, China) and verified by DNA sequencing. The confirmed fragment in *pEASY*-T1vector was digested with KpnI and SacI for subsequent ligation to the modified p1301 (http://www.cambia.org) vector driven by the 35S promoter. To generate the *RFC1* RNAi plants, the 334 bp *RFC1* specific fragment upstream of the AAA domain was amplified using primers oMF2082 and oMF2083 with restriction sites NcoI/Xbal and ApaI/SalI, respectively. The PCR product was first cloned into vector p*EASY*-T1 Simple (Transgen, Beijing, China) for verification by sequencing. The sense and antisense fragment digested by NcoI-ApaI and SalI-Xbal respectively were cloned into the same sites in pMeioDMC1-Intron sequentially. Both constructs were introduced into *Agrobacterium tumefaciens* GV3101 for transforming the heterozygous *rfc1-2* plant lines and wild type, respectively, using a floral-dip method [65]. Positive T1 plants were screened on 0.5×MS medium containing 25 mg/L hygromycin and transferred to soil in greenhouse under 22°C, 16 h light/8 h dark.

### Fluorescence *in situ* hybridization (FISH)

Chromosome spreads were performed as described (Ross *et al.*, 1996). Slides with chromosome preparations were dehydrated with an ethanol series (70%, 90% and 100%) prior to being used for FISH. The 180 bp repetitive sequence of centromere was described previously [66]. The telomere (clone pAtT4) [67], 5S rDNA [68], 45S rDNA [68], and two BAC clones (F19K16 and F1N21) located on the chromosome 1 upper arm were labeled as probes according to the protocol provided by Nick Translation Kit (No: 11570013910 Roche Diagnostics, Indianapolis, IN, USA). FISH analysis was conducted according to a published procedure [69]–[70]. Anti-DIG-rhodamine Fab fragment (Cat#1207750 Roche Diagnostics) was used as second antibody to detect probe labeled with DIG. Chromosomes were counterstained with 4,6-diamidinophenylindole (DAPI) (Vector Laboratories, Burlingame, CA, USA). Chromosome images were captured under the Zeiss Axio Imager A2 fluorescence microscope with a high resolution microscopy camera AxioCam MRc Rev. 3 FireWire (D).

### Generation of polyclonal antibodies

Peptide antisera were raised in rabbits against the amino acid sequence of NAVQQQDDEETQHGPF (AtRAD51), EREENDEDEDLFEMIDK (AtDMC1), NCSQASQ DRRGRKTS (AtASY1), GSKRSEHIRVRSDNDNVQD (AtZYP1), GSSGSRKAAGKGRG RGK (AtRFC1) conjugated to KLH (GL Biochem, Shanghai, Ltd: www.glschina.com). Western blot was performed to verify the individual antigen for antibodies using the total *Arabidopsis* inflorescence proteins (data not shown). Specificity of the purified anti-RAD51 and DMC1 antibodies was confirmed using the *rad51, dmc1* and *rfc1* mutants as negative controls.

The specificity of the anti-RFC1 antibodies was verified using a western with the immunizing peptide as a competitive antigen, according to a previously described procedure (Blocking with Immunizing Peptide (BL) Protocol: www.abcam.com/technical).

### Immunolocalization

Immunofluorescence was performed as previously described with minor modifications [71]. The primary antibodies with diluted 1∶100 (ASY1, RAD51 and DMC1) or 1∶200 (ZYP1) in blocking buffer were added to the slides covered with parafilm and incubated overnight at 4°C in a moisture chamber. The slides were washed with washing buffer II (1×PBS+0.1% Tween 20) three times and 15 min for each time. The secondary antibody (goat anti-rabbit (H+L), lot: 835724, Invitrogen, Foster City, CA, USA), with 1∶500 dilution in blocking buffer was added to slides, covered with parafilm and incubated in humidified atmosphere at 37°C for 60 min in the dark. The slides were washed with washing buffer II three times for 15 min and mounted in vectashield antifade medium (Vector Laboratories, Burlingame, CA, USA) with 1.5 µg/ml DAPI. Images were taken using an AxioCam HRc (Zeiss) camera.

### Measurement of CO frequencies

*rfc1-2* ^−/−^ plants were crossed to lines carrying transgenic markers (M) encoding the fluorescent protein (dsRED2, eYFP and/or CFP) expressed from the post-meiotic pollen-specific promoter *LAT52* in a *qrt1-2^−/−^* background. F2 *rfc1-2* ^−/−^ M^−/+^ plants were selected by monitoring pollen fluorescence according to a previous procedure [36]. Pollen grains were scored as recombinant or parental by monitoring the expression of the fluorescent markers. CO frequencies between any two transgenic markers or double CO frequencies in adjacent intervals were calculated using the following formula: (recombinant pollen/total viable pollen grains)*100%. All photographs were taken using the Zeiss Axio Imager A2 fluorescence microscope with a high resolution microscopy camera AxioCam MRc Rev. 3 FireWire (D).

### Statistical methods

All of the data were analyzed statistically using Microsoft Excel 2007 (Microsoft) for calculating mean and SE and T-test. The reported P values are either exact values or Gaussian approximations.

### Accession numbers

*Arabidopsis* Genome Initiative gene identifiers are as follows: RFC1 (At5g22010); SPO11 (At2g13170); RAD51 (At5g20850); MSH4 (At4g17380); MUS81 (At4g30870); PTD (At1g12790).

## Supporting Information

Figure S1 Molecular characterization of *rfc1* alleles and phenotype of *rfc1-1.* (A) An illustration of the *RFC1* gene structure. White, block boxes and dashes represent the untranslated region (UTR), coding regions of exons and introns, respectively. *rfc1-1* is a G to A point mutation. The *rfc1-2* and *rfc1-3* alleles are T-DNA insertional lines. The peptide position (from 919 to 935aa) was designed to generate polyclonal antibodies. F1/R1, F2/R2 and F3/R3 refer to primers. The RFC1 protein has three major domains: BRCT, AAA and RFC. (B) Molecular characterization of the *rfc1-2* allele. F1 and R1 are the *RFC1*-specific primers spanning the T-DNA insertional site. LBb1.3 is a primer for the T-DNA left board. (C) RT-PCR analysis of the *RFC1* expression upstream of, spanning, and downstream of T-DNA insertional site in wild type and *rfc1-2*. (D) Western blot analysis of the intact RFC1 protein in wild type and *rfc1-2* (upper panel). No band was detected when a similar Western blot experiment was performed in the presence of the immunizing RFC1 peptide, along with the RFC1 antibody, indicating that the anti-RFC1 antibodies were specific. (E) The *rfc1-1* mutant showed an obvious reduction of the number of viable pollen grains with dead pollen grains stained in green. Chromosome behavior of *rfc1-1* at pachytene (F), diakinesis (G) and metaphase I (H).
(TIF)

Figure S2 Phenotypes of the *rfc1-2* rescued plants, *RFC1-RNAi* transgenic plants and *rfc1-2/rfc1-3* trans-heterozygous plants. A rescued plant showing normal fertility and meiosis, including viable pollen grains (A), the fully synapsed chromosomes at pachytene (B), the five bivalents at diakinesis (C), and chromosomes well aligned near the equator at metaphase I (D). The *rfc1-2/rfc1-3* trans-heterozygous plants with few viable pollen grains (E), pachytene chromosomes with “bubble” (F, arrow), the presence of multivalents at diakinesis (G) and the interaction between non-homologs at metaphase I (H, arrow). A *ProDMC1*-*RFC1-RNAi* transgenic plant with few viable pollen grains (I), pachytene chromosomes with “bubble” (J, arrow), and multivalents at diakinesis (K) and the interaction between non-homologs at metaphase I (L, arrow), consistent with meiotic phenotypes of the *rfc1-2* single mutant and *rfc1-2/rfc1-3* trans-heterozygous plants. Bar, (B–D, F–H, J–L) 10 µm; (A, E and I) 500 µm.
(TIF)

Figure S3 FISH analysis of wild type and *rfc1-2.* (A) Wild type and (C) *rfc1-2* with two BAC F9K16 signals at zygotene (arrow); wild type had two signals close to each other at diplotene (B, arrow), but *rfc1-2* had two separated signals (D, arrow). (E) *rfc1-2* with separated pachytene chromosome 1 arms showing two BAC (F9K16) signals (arrow). (F) *rfc1-2* with two F9K16 signals on the pachytene chromosomes with “bubble” (arrow). (G and H) Enlarged area of E and F. (I–K, O–Q) The 45S rDNA signals were similar between wild type and mutant. (I1 and O1) leptotene, (J1 and P1) zygotene, (K1 and Q1) pachytene. At metaphase I, wild type had four signals on two bivalents (L1), but the mutant showed more than 4 signals (R1), suggesting that non-homologs were associated. Compared to wild type with equally chromosome segregation during meiosis I and II (M1 and N1), *rfc1-2* showed unequal chromosome segregation (S1 and T1). Bar, 10 µm.
(TIF)

Figure S4 DMC1 localization in wild type and *rfc1-2.* (A and E) leptotene, (B and F) zygotene, (C and G) pachytene, (H) An enlarged region at pachytene, (D) the *dmc1* mutant at pachytene. The *rfc1-2* mutant showed similar number of DMC1 foci to wild type before pachytene, but late pachytene cells had overlapped DMC1 signals with chromosomes (G), unlike the wild type with punctate DMC1 distribution on chromosome (C), the mutant had longer stretches of DMC1 signals. In each row of three panels, the left panel shows blue colored chromosomes strained with DAPI; the middle panel shows red colored signals for proteins as indicated above the panel; and the right panel shows the merged image of DAPI and protein signals. Bar, 10 µm.
(TIF)

Table S1 Genetic transmission of the *rfc1-2* mutant.
(DOC)

Table S2 Observed pollen with and without fluorescence in wild type and the *rfc1-2* mutant.
(DOC)

Table S3 Primers used in this study.
(DOC)
