# Supplementary figures and images for: The DNA Replication Factor RFC1 Is Required for Interference-Sensitive Meiotic Crossovers in Arabidopsis thaliana
Source: PLoS Genet. 2012 Nov 8;8(11):e1003039. doi: 10.1371/journal.pgen.1003039 (PMC3493451; doi:10.1371/journal.pgen.1003039)

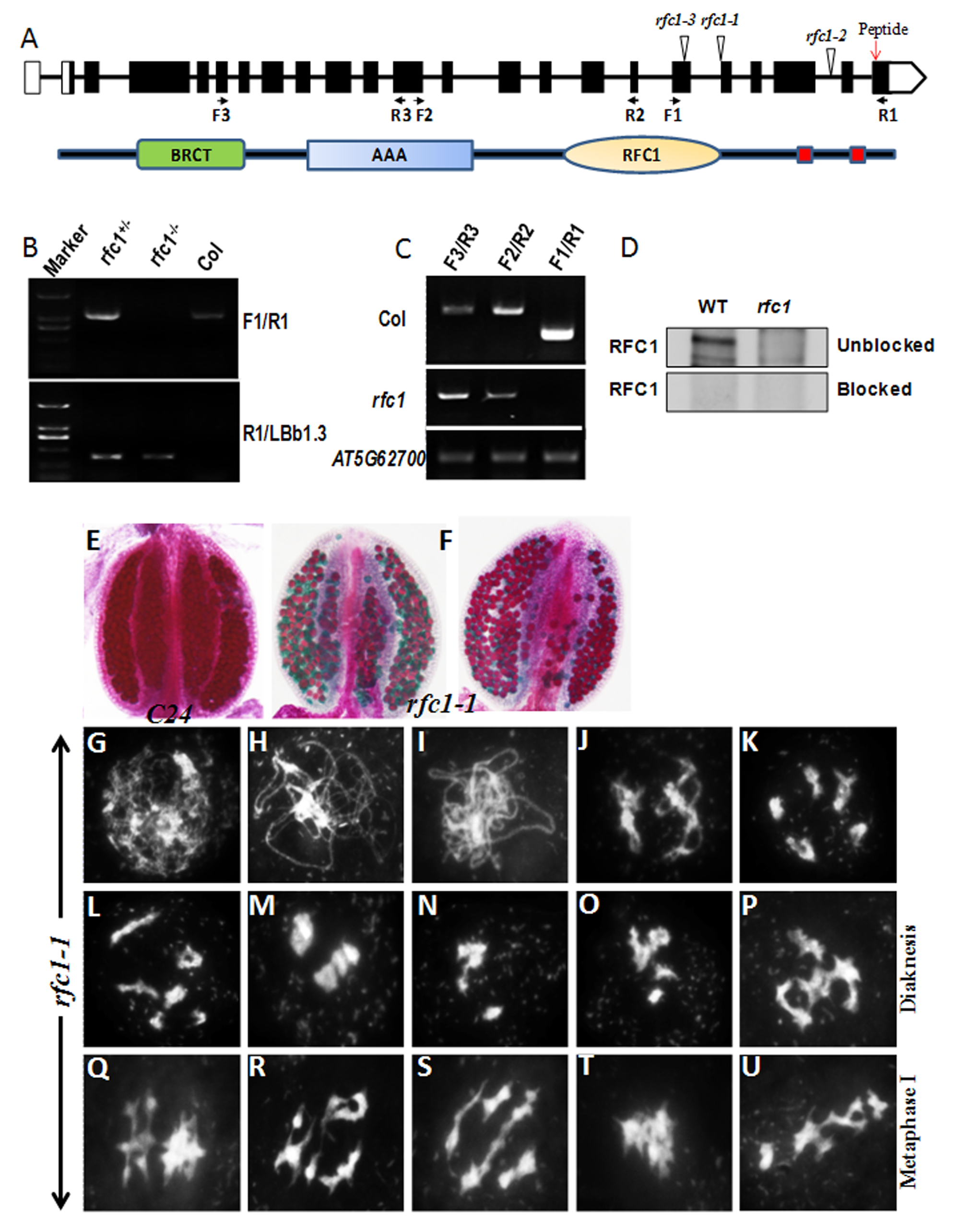

Supplement: Figure S1 — Molecular characterization of rfc1 alleles and phenotype of rfc1-1. (A) An illustration of the RFC1 gene structure. White, block boxes and dashes represent the untranslated region (UTR), coding regions of exons and introns, respectively. rfc1-1 is a G to A point mutation. The rfc1-2 and rfc1-3 alleles are T-DNA insertional lines. The peptide position (from 919 to 935aa) was designed to generate polyclonal antibodies. F1/R1, F2/R2 and F3/R3 refer to primers. The RFC1 protein has three major domains: BRCT, AAA and RFC. (B) Molecular characterization of the rfc1-2 allele. F1 and R1 are the RFC1-specific primers spanning the T-DNA insertional site. LBb1.3 is a primer for the T-DNA left board. (C) RT-PCR analysis of the RFC1 expression upstream of, spanning, and downstream of T-DNA insertional site in wild type and rfc1-2. (D) Western blot analysis of the intact RFC1 protein in wild type and rfc1-2 (upper panel). No band was detected when a similar Western blot experiment was performed in the presence of the immunizing RFC1 peptide, along with the RFC1 antibody, indicating that the anti-RFC1 antibodies were specific. (E) The rfc1-1 mutant showed an obvious reduction of the number of viable pollen grains with dead pollen grains stained in green. Chromosome behavior of rfc1-1 at pachytene (F), diakinesis (G) and metaphase I (H). (TIF) [file pgen.1003039.s001.tif]

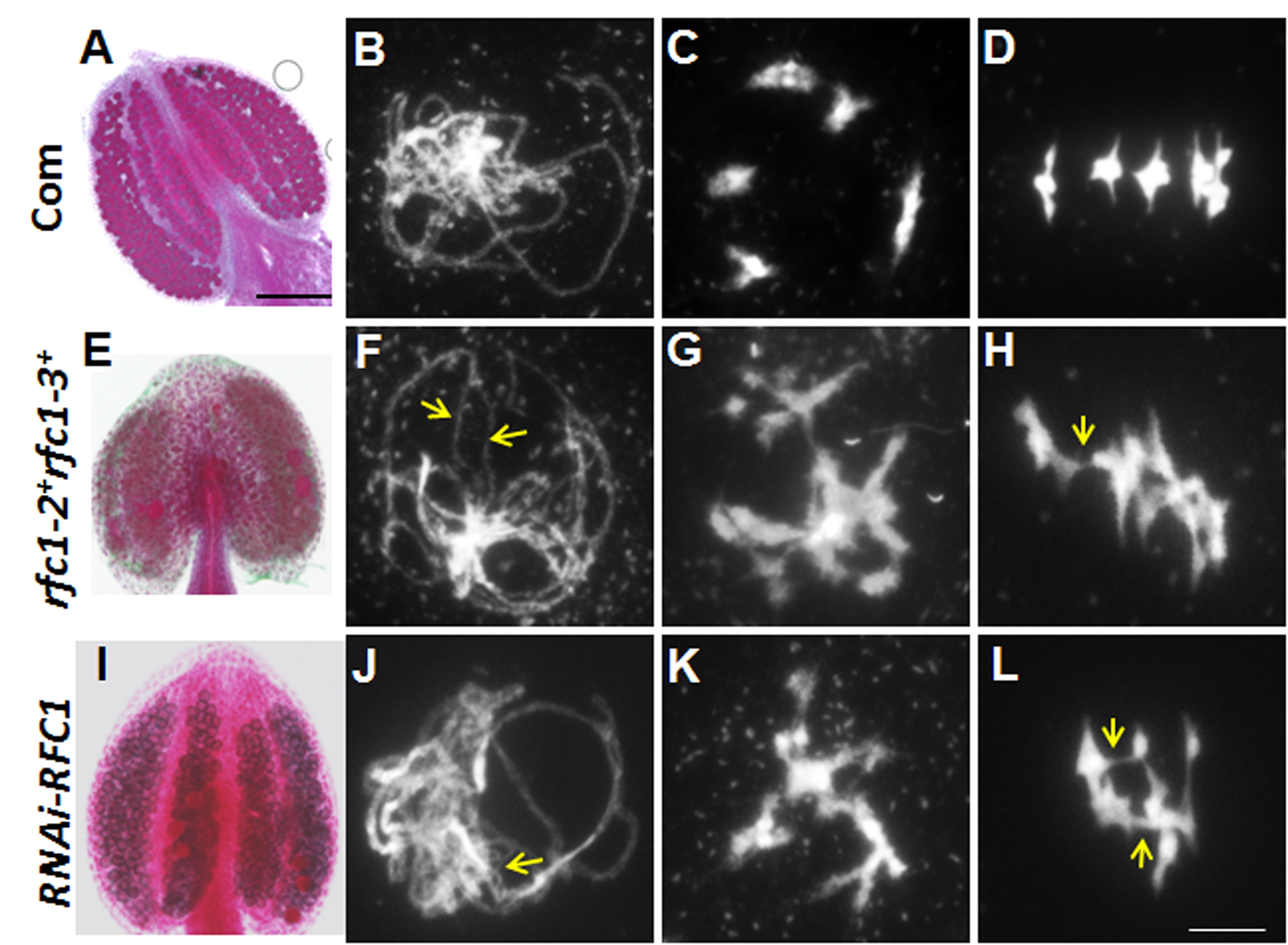

Supplement: Figure S2 — Phenotypes of the rfc1-2 rescued plants, RFC1-RNAi transgenic plants and rfc1-2/rfc1-3 trans-heterozygous plants. A rescued plant showing normal fertility and meiosis, including viable pollen grains (A), the fully synapsed chromosomes at pachytene (B), the five bivalents at diakinesis (C), and chromosomes well aligned near the equator at metaphase I (D). The rfc1-2/rfc1-3 trans-heterozygous plants with few viable pollen grains (E), pachytene chromosomes with “bubble” (F, arrow), the presence of multivalents at diakinesis (G) and the interaction between non-homologs at metaphase I (H, arrow). A ProDMC1-RFC1-RNAi transgenic plant with few viable pollen grains (I), pachytene chromosomes with “bubble” (J, arrow), and multivalents at diakinesis (K) and the interaction between non-homologs at metaphase I (L, arrow), consistent with meiotic phenotypes of the rfc1-2 single mutant and rfc1-2/rfc1-3 trans-heterozygous plants. Bar, (B–D, F–H, J–L) 10 µm; (A, E and I) 500 µm. (TIF) [file pgen.1003039.s002.tif]

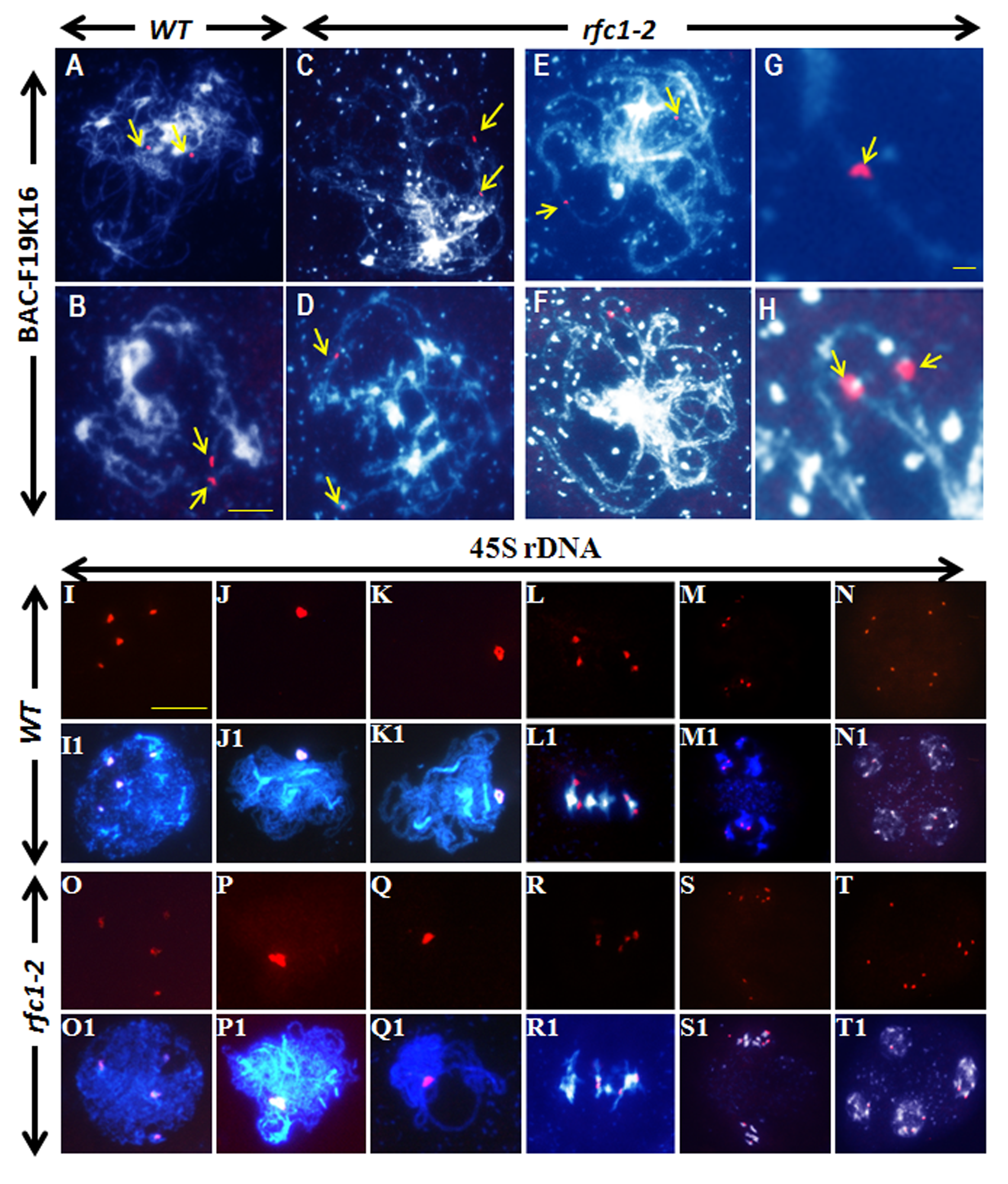

Supplement: Figure S3 — FISH analysis of wild type and rfc1-2. (A) Wild type and (C) rfc1-2 with two BAC F9K16 signals at zygotene (arrow); wild type had two signals close to each other at diplotene (B, arrow), but rfc1-2 had two separated signals (D, arrow). (E) rfc1-2 with separated pachytene chromosome 1 arms showing two BAC (F9K16) signals (arrow). (F) rfc1-2 with two F9K16 signals on the pachytene chromosomes with “bubble” (arrow). (G and H) Enlarged area of E and F. (I–K, O–Q) The 45S rDNA signals were similar between wild type and mutant. (I1 and O1) leptotene, (J1 and P1) zygotene, (K1 and Q1) pachytene. At metaphase I, wild type had four signals on two bivalents (L1), but the mutant showed more than 4 signals (R1), suggesting that non-homologs were associated. Compared to wild type with equally chromosome segregation during meiosis I and II (M1 and N1), rfc1-2 showed unequal chromosome segregation (S1 and T1). Bar, 10 µm. (TIF) [file pgen.1003039.s003.tif]

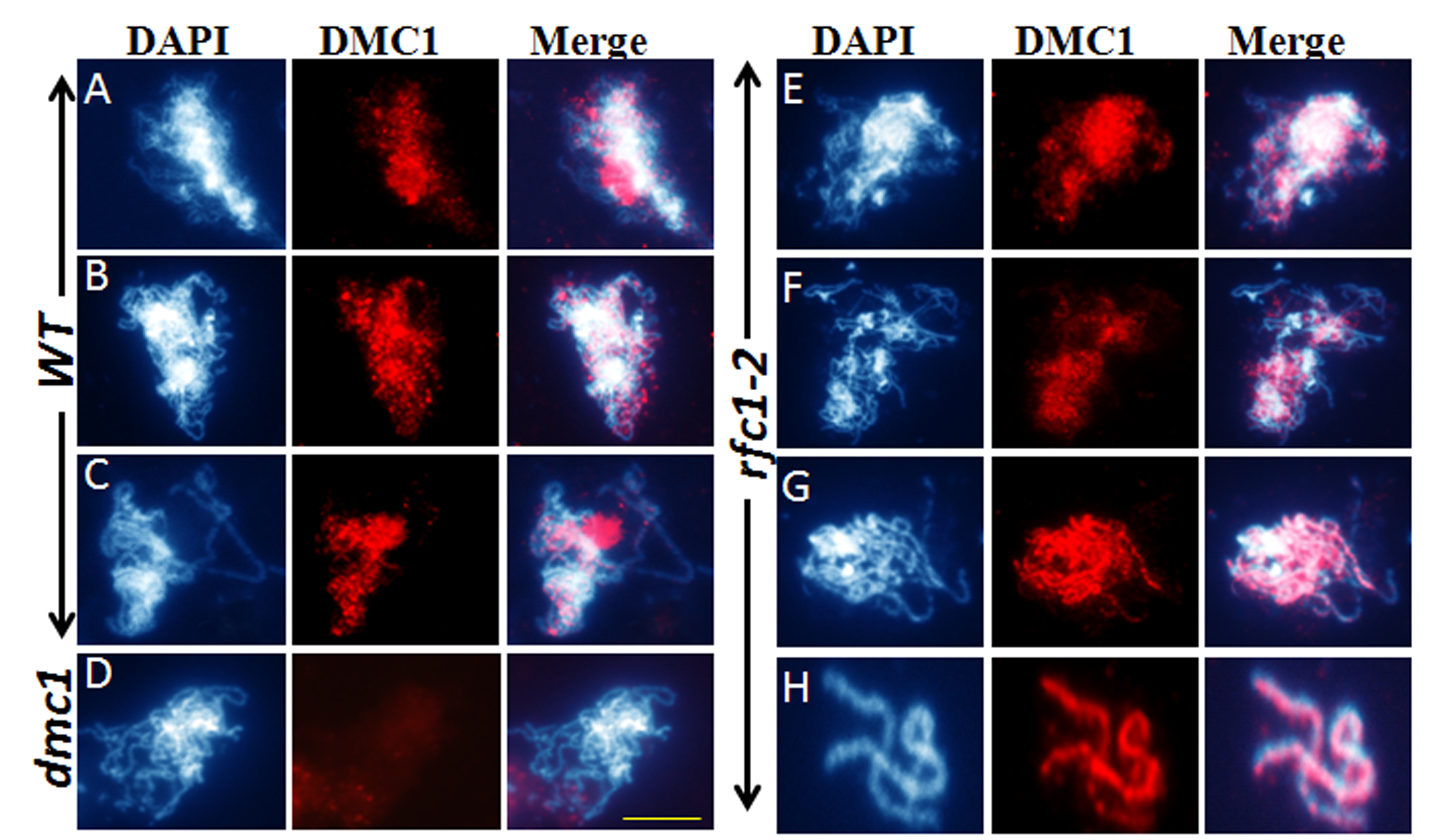

Supplement: Figure S4 — DMC1 localization in wild type and rfc1-2. (A and E) leptotene, (B and F) zygotene, (C and G) pachytene, (H) An enlarged region at pachytene, (D) the dmc1 mutant at pachytene. The rfc1-2 mutant showed similar number of DMC1 foci to wild type before pachytene, but late pachytene cells had overlapped DMC1 signals with chromosomes (G), unlike the wild type with punctate DMC1 distribution on chromosome (C), the mutant had longer stretches of DMC1 signals. In each row of three panels, the left panel shows blue colored chromosomes strained with DAPI; the middle panel shows red colored signals for proteins as indicated above the panel; and the right panel shows the merged image of DAPI and protein signals. Bar, 10 µm. (TIF) [file pgen.1003039.s004.tif]
