# Supplementary material for: The DNA Replication Factor RFC1 Is Required for Interference-Sensitive Meiotic Crossovers in Arabidopsis thaliana
Source: PLoS Genet. 2012 Nov 8;8(11):e1003039. doi: 10.1371/journal.pgen.1003039 (PMC3493451; doi:10.1371/journal.pgen.1003039)
Supplement: Table S1 — Genetic transmission of the rfc1-2 mutant. (DOC) [file pgen.1003039.s005.doc]

**Table S1 Genetic transmission of the *rfc1-2* mutant**

| *rfc1*-/+ selfing | | | *rfc1*-/+ ×WT | | |  | WT×*rfc1*-/+ | | | |
| --- | --- | --- | --- | --- | --- | --- | --- | --- | --- | --- |
| Kan^R^/Kan^S^ seedings | Kan^R^/Kan^S^ rations | x^2^ | Kan^R^/Kan^S^ seedings | Kan^R^/Kan^S^ rations | x^2^ | TE_f_ | Kan^R^/Kan^S^ seedings | Kan^R^/Kan^S^ rations | x^2^ | TE_m_ |
| 96:38 | 2.53 | 0.81 | 293:284 | 1.03 | 0.14 | 100% | 105:224 | 0.47 | 43.04 | 47% |

Kan^R^, kanamycin-resistant seedlings; Kan^S^, kanamycin-sensitive seedlings; TE, transmission efficiency = (Kan^R^/Kan^S^)×100%. χ², chi-square test.
