# Supplementary material for: The DNA Replication Factor RFC1 Is Required for Interference-Sensitive Meiotic Crossovers in Arabidopsis thaliana
Source: PLoS Genet. 2012 Nov 8;8(11):e1003039. doi: 10.1371/journal.pgen.1003039 (PMC3493451; doi:10.1371/journal.pgen.1003039)
Supplement: Table S2 — Observed pollen with and without fluorescence in wild type and the rfc1-2 mutant. (DOC) [file pgen.1003039.s006.doc]

**Table S2 Observed pollen with and without fluorescence in wild type and the *rfc1-2* mutant**

| **Genotype** | **RYC fluo PG** | **No fluo PG** | **RY fluo PG** | **C fluo PG** | **YC fluo PG** | **R fluo PG** | **RC fluo PG** | **Y fluo PG** | **Total PG** |
| --- | --- | --- | --- | --- | --- | --- | --- | --- | --- |
| **WT** | 5592 | 5591 | 148 | 149 | 284 | 285 | 1 | 2 | 12052 |
| ***rfc1-2*** | 693 | 1114 | 98 | 41 | 111 | 232 | 17 | 14 | 2320 |
| **WT** | / | 2633 | / | 731 | / | 731 | 2633 | / | 6728 |
| ***rfc1-2*** | / | 991 | / | 168 | / | 212 | 628 | / | 1999 |

RYC fluo PG represents the red, yellow, cyan fluorescent pollen grains; RY fluo PG represents the red, yellow fluorescent pollen grains and so on.
