# Supplementary material for: The DNA Replication Factor RFC1 Is Required for Interference-Sensitive Meiotic Crossovers in Arabidopsis thaliana
Source: PLoS Genet. 2012 Nov 8;8(11):e1003039. doi: 10.1371/journal.pgen.1003039 (PMC3493451; doi:10.1371/journal.pgen.1003039)
Supplement: Table S3 — Primers used in this study. (DOC) [file pgen.1003039.s007.doc]

**Table S3 Primers used in this study**

| Name | Number | Sequences |
| --- | --- | --- |
| *rfc1-1*  genotyping | oMF2169 | 5’-TTAAATGGCAGGCATTCTGAG-3’ |
|  | oMF2170 | 5’-ACACGATTTCCTTTTTCATGG-3’ |
|  | oMF270 | 5’-ATTTTGCCGATTTCGGAAC-3’ |
| *rfc1-2 genotyping* | oMF1876 | 5’- TTCCAGCATAGCAGCTATTCG-3’ |
|  | oMF1877 | 5’-GGATCAAGGGAGGTACTCGAG-3’ |
| *spo11-1* genotyping | oMF2520 | 5’-ACGTATCGGGCCTAAATTCC-3’ |
|  | oMF-2521 | 5’-TTTGGAGATCTTCCTTCAGCC-3’ |
|  | oMF2522 | 5’-ACTGGGATTCGTCTTGGACA-3’ |
| *rad51*  genotyping | oMF1895 | 5’-TTCAGGATGGTGTCTCAGAGC-3’ |
|  | oMF1896 | 5’-ATGCCAAGGTTGACAAGATTG-3’ |
|  | oMF1716 | 5’-GCTTCCTATTATATCTTCCCAAATTACCAATACA-3’ |
| *mus81* genotyping | oMF2090 | 5’-TTAACCATCCACTTGCCAAAG-3’ |
|  | oMF2091 | 5’-CCCAAAGATGAACCAAGTGAC-3’ |
| *msh4*  genotyping | oMF2096 | 5’-GAGATCTGTTGCTGAGGAACG-3’ |
|  | oMF2097 | 5’-GTTTCAACTGCGAGATGGAAC-3’ |
| *ptd*  genotyping | oMF1593 | 5’-GACAAATCTGATGAAGATGTTTG-3’ |
|  | oMF1594 | 5’-TGCTTATCTGTGTTGTCGGAAAT-3’ |
| Complementation  construct | oMF1000 | 5’-AAAGGTACCATGTCGGATATTAGGAAGTGGT-3’ |
|  | oMF1001 | 5’-AAAGAGCTCTCTCTTTCTCTTGGCACCAG-3’ |
| RNAi construct | oMF2082 | 5’-GTCTAGA CCATGGCGACAGGGGAGGTGTTGC-3’ |
|  | oMF2083 | 5’-CTTGTCGAC GGGCCCTGCTTAGCAGGCGCTGCCTG-3’ |
| RFC1 expression  RT-PCR | oMF1368 | 5’-CCTGATTGCTTAGCTGGATTG-3’ |
|  | oMF1369 | 5’-CATGAACATGCAGATCCTCC-3’ |
|  | oMF1370 | 5’-GGACGTGAAAATCCGATGGA-3’ |
|  | oMF1371 | 5’-CTCTTTCTCTTGGCACCAGA-3’ |
|  | oMF2029 | 5’-GCTTTGACCTATCGAAGTTGG-3’ |
|  | oMF2030 | 5’-GTAGATGGAATGTCTGCAGG-3’ |
